# Ownership reform and the changing manufacturing landscape in Chinese cities: The case of Wuxi

**DOI:** 10.1371/journal.pone.0173607

**Published:** 2017-03-09

**Authors:** Lei Zhou, Shan Yang, Shuguang Wang, Liyang Xiong

**Affiliations:** 1 School of Geographic and Biologic Information, Nanjing University of Posts and Telecommunications, Nanjing, China; 2 School of Geography, Nanjing Normal University, Nanjing, China; 3 Department of Geography, Ryerson University, Toronto, Canada; 4 Department of Geography, University of Wisconsin-Madison, Madison, Wisconsin, United States of America; 5 Jiangsu Center for Collaborative Innovation in Geographical Information Resource Development and Application, Nanjing, China; Peking University, CHINA

## Abstract

Since the economic transition, manufacturing in China has undergone profound changes not only in number of enterprises, but also in ownership structure and intra-urban spatial distribution. Investigating the changing manufacturing landscape from the perspective of ownership structure is critical to a deep understanding of the changing role of market and government in re-shaping manufacturing location behavior. Through a case study of Wuxi, a city experiencing comprehensive ownership reform, this paper presents a detailed analysis of the intra-urban spatial shift of manufacturing, identifies the location discrepancies, and examines the underlying forces responsible for the geographical differentiations. Through zone- and district-based analysis, a distinctive trend of decentralization and suburbanization, as well as an uneven distribution of manufacturing, is unveiled. The results of Location Quotient analysis show that the distribution of manufacturing by ownership exhibits distinctive spatial patterns, which is characterized by a historically-based, market-led, and institutionally-created spatial variation. By employing Hot Spot analysis, the role of development zones in attracting manufacturing enterprises of different ownerships is established. Overall, the location behavior of the diversified manufacturing has been increasingly based on the forces of market since the land marketization began. A proactive role played by local governments has also guided the enterprise location decision through spatial planning and regulatory policies.

## 1. Introduction

The economic transition in the past three decades has stimulated the rapid development of China’s manufacturing industry. Before the economic transition began in 1978, manufacturing in China was a simple yet rigid production system, which was either state-owned or collectively-owned with a limited number of factories. Since 1978, manufacturing in China has undergone profound changes: the number of plants increased dramatically; ownership diversified considerably; foreign-invested enterprises (FIEs) were allowed to set up factories; and more importantly, non-state owned domestic enterprises have become an important part of the national economy. In essence, manufacturing has moved away from a centrally planned system towards a market-oriented industry.

Manufacturing activities, which constitute a large proportion of urban economy, occupy large tracts of urban land. Under the economic transition, the spatial distribution of manufacturing in Chinese cities has attracted considerable scholarly attention. Existing studies mainly focused on the changing intra-urban manufacturing locations and the driving forces [1–2], location characteristics and the influencing factors of a particular manufacturing sector [3–5], or a particular capital source, particularly foreign direct investment (FDI) [6–9]. In general, Chinese cities have experienced a decentralization of industrial activities from the city center, accompanied with the formation of new industrial agglomerations in a variety of suburban development zones [2]. The prevailing view is that the intra-urban location behavior of manufacturing in China has become increasingly attributed to market forces, rather than to the socialist ideology that played a significant role before the economic reform [10]. In a sense, this reflects a convergence towards the advanced capitalist economies, where intra-urban distribution of manufacturing is intrinsically under the forces of market mechanism [11]. However, some scholars argue that active interventions by local municipalities through a range of policy instruments relating to industrial location have also affected the spatial process of intra-urban manufacturing migration [8].

Although the ownership structure of manufacturing in China has changed significantly, there is a lack of studies analyzing the changing intra-urban manufacturing landscape from the perspective of ownership reform, leaving a research gap to be filled. In China, ownership of an enterprise represents its operating mechanism, government-enterprise relationship, and even government interventions of the enterprise [12], which in turn can affect the enterprise’s location behavior. Also, land use regulations, which reflect the effects of both government policies and market forces, play a particularly important yet often overlooked role in shaping the changing manufacturing landscape at the intra-urban scale [2, 13]. Therefore, we assume that substantial intra-urban location variations exist among manufacturing activities of different ownership, and that the land use reform, which started in 1987 and moved away from free use to paid use, significantly altered the intra-urban location decisions of manufacturing activities.

Furthermore, existing research on intra-urban industrial location patterns in Chinese cites is confined mainly to Beijing, Shanghai, Suzhou, Wenzhou, Guangzhou. With few exceptions [14], little attention has been paid to other important urban centers, such as Wuxi—an advanced manufacturing base in the Shanghai-centered Yangtze River Delta (YRD), and a representative city of the Sunan Model in China [14] with a high level of marketization and globalization. The Sunan Model was originated in the 1980s, which attributes the post-reform development of Sunan (Southern Jiangsu Province) to the local municipality-directed collectively-owned township and village enterprises (TVEs). Since the early 1990s, however, with deepening reforms, Sunan has moved “beyond the Sunan Model” through privatization and internationalization [14]. Neither like Suzhou (a leading city in Sunan characterized with FDI-driven development), nor like Wenzhou (marked by the development of POEs and dubbed as the well-known “Wenzhou Model”), the economy of Wuxi is dominated by small-sized POEs as well as large-scale FIEs, showing a particular trajectory of the transformation of the Sunan Model. Research on the changing manufacturing landscape in Wuxi is important because its diversified ownership structure provides a typical case for a detailed analysis of location differentiations among manufacturing of different ownership types, and for an investigation the changing role of market and government in shaping the changing manufacturing landscape. Through questionnaire surveys and interviews with firm management and government officials, Yuan et al [14] conducted research on the industrial location and transition in Wuxi against the Sunan Model. While that study advanced our understanding of the changes in enterprise structure and location, it is limited to one central city district—Nanchang District, whose manufacturing ownership is dominated by privately-owned enterprises (POEs), and the surveyed firms are generally small in size. A single urban district in the central city cannot fully represent the ownership restructuring in the expansive Wuxi city. Therefore, there is still a need to take Wuxi as a study area to explore the changing landscape of manufacturing of different ownership types.

Through the case study of Wuxi, we aim to achieve two research objectives. First, we identify and analyze the intra-urban manufacturing location shifts and the spatial variations by ownership type. Second, we examine the underlying forces that have re-shaped the manufacturing landscape in Wuxi. The specific questions to be addressed are: (1) to what extent, the spatial differentiation of manufacturing is attributed to the force of land marketization? (2) what are the roles played by different levels of government in the current distribution of manufacturing plants, and how did the role of governments change during the economic transition? Specifically, we hypothesize that development zones play a critical role in reshaping the manufacturing landscape in Wuxi through their preferential policies, and that enterprises that have high rent-biding abilities tend to congregate in the high-level development zones that have complete infrastructures but also charge higher rent.

## 2. Theoretical framework and research context

### 2.1 Advances in manufacturing location theories

Previous research reveals that manufacturing location selection is by no means random [6]. Classical location theories focused on economic factors, and conceptualized enterprise location behavior mainly from the perspectives of cost reduction (transport costs, wage, and land price) and agglomeration economies [15]. Nowadays the list of important locational determinants has been expanded to include labor skills, physical infrastructure, and institutional factors. Particularly, with the development of new institutional economics, increasing attention has been paid to the role of government interventions in affecting manufacturing site selection [16–17]. Government interventions, including tax incentives, subsidies, regulations, and other legal instruments [16], sometimes are intended to redress a market failure [18]. Provision of direct governmental economic aid, tax benefits or subsidies to industries is effective in attracting them to locate in specified areas. Conversely, higher tax rate for a particular sector appears to be of some significance in deterring them to aggregate. Recently, governments have also extended the range, and increased the effectiveness, of their policy instruments relating to the protection of environment, which presents a significant new influence on the location of pollution intensive manufacturing [19].

Since the mid-twentieth century, as the massive decentralization of industry has proceeded at an accelerating pace in North America and Western Europe, increasing attention has been paid to analyzing the industrial locational factors at the municipal level. Several factors are identified for contributing to an explanation of the new intra-urban manufacturing geography [5, 11, 20–21]: pressure by land market, problems of access and transport, deterioration of available real estate, changes in the structure of industrial concerns, government planning restrictions, and, to a lesser extent, agglomeration economies.

Besides external environment, the attribute of the enterprise itself also plays a significant role in the location decision-making of manufacturing. This can be verified by the distinctive location behaviors exhibited by enterprises of different types. For High-tech industries, a highly skilled labor force is particularly important [22]. Lejpras & Stephan [23] found that proximity to local research institutes and universities is the most important location characteristic of high-tech firms. As the outcome of globalization, FIEs also show unique location behavior. In recent years, the location selections of FIEs are increasingly affected by “created assets” [17], including knowledge-based assets, market size, infrastructure and institutions of the host economy. Particularly, FIEs are positively related to the quality of formal institutions, which is usually embodied in the policy incentives of the development zone. That is, the location of development zones largely defines the spatial pattern of FIEs [24–26].

Manufacturing location is also constrained by wider social, political and economic context [27]. Economic transition in China has induced a significant change of the intra-urban manufacturing landscape and provided a valuable opportunity to explore the impact of market forces and government interventions on manufacturing location because the entire set of formal institutions has been remodeled [17]. A distinct yet diverse economic environment has evolved, as the institutions reflect both the heritage of the planned system the characteristic of the market economy, which makes the intra-urban manufacturing location selection mechanism more complicated.

Since the economic transition, traditional location factors, such as land price and transport cost, are playing an increasingly significant role in the location decision of manufacturing [2]. Moreover, the role of formal institutional arrangement of the local government (such as manufacturing retreat in favor of tertiary sector activity expansion, and the establishment of development zones) attracted considerable scholarly attention [1]. Researchers also analyze the government interventions on the distribution of high-tech and pollution intensive manufacturing [1, 4]. In addition, through a large number of case studies of FIEs, some valuable conclusions of their location behavior have been drawn. Generally, FDI locations within Chinese cities are significantly impacted by development zones, accessibility to local transportation facilities, and the availability of industrial land and real estate [5–6, 9, 28–29].

### 2.2 Research context and conceptual framework

**Fig 1:**
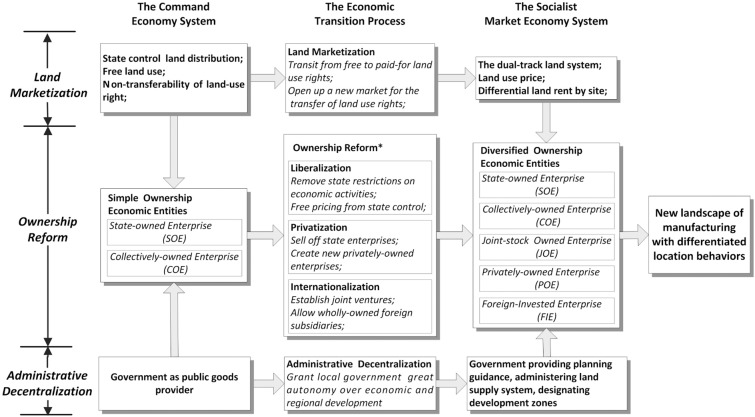
Conceptual framework for analyzing the new landscape of manufacturing in China (Note: * Part B: ownership reform is modified from [30–31]).

In this paper, we follow the perspective of enterprise ownership to explore the changing role of land market and government in shaping the new intra-urban manufacturing landscape under economic transition. Specifically, land marketization, ownership reform, and administrative decentralization together form the conceptual framework for this study (Fig 1).

#### 2.2.1 Land marketization (Part A in Fig 1)

In the Western capitalist countries, the price of urban land typically declines systematically from the city center outwards to the suburbs. All other things being equal, high land rents at the city center tend to deter industry, while low land rents at the city periphery tend to attract industry [11]. Yet, this was totally different in the pre-reform China.

Based on the ideology that all land was common property, urban land in China was nationalized after 1949 [32]. In the command economy, land was nominally worthless and taken as a means of production rather than a commodity subject to market transaction [33]. Each enterprise was allocated a piece or tract of land by the state free of charge. The location and amount of the land allocated to an enterprise depended on its political affiliation with the government and the political environment in which socioeconomic functions and production were planned and organized [2]. Enterprises were passive takers of state orders, and production location decisions were not informed by calculations of comparative advantage [34]. Due to low levels of industrialization, land supply and price seldom became the core elements to be considered in the industrial layout.

In order to improve land use efficiency and to meet the demand of foreign investors for clarification of land property rights, China in 1987 started to reform its land use system [33]. The new land use rights system, literally the “pay for transfer of land-use rights”, was made official by an amendment to China’s Constitution in 1988 [32]. Since then, the land use system reform has gradually been carried forward from free allocation to paid use, and to the highest bidders through the market mechanism.

However, inheriting from the command economy system, the current land use system is actually a dual-track land disposition system, in which free administrative allocation of land use rights coexists with paid transfer. On the one hand, the state-owned enterprises (SOEs) still receive land use rights through the “plan track” of administrative allocation, by paying a low and symbolic fee. On the other hand, commercial users obtain land use rights through the “market track” of transfer that requires them to pay a much higher price determined by the market value of the land [33].

#### 2.2.2 Ownership reform resulting from liberalization, privatization and internationalization (Part B in Fig 1)

The post-socialist states, of which China is one, are often referred to as transitional economies [31], during which the most fundamental formal institutional reform is the ownership transition of economic entities [35]. Based on the experience of the post-socialist countries, Bradshaw (1996) theorized a model of ownership transition. While not manufacturing specific, this model provides a useful framework for examining the manufacturing ownership transition in China. In this theorization, Bradshaw identifies three dimensions in the ownership reform process.

Economic liberalization refers to the gradual removal of government restrictions on economic activities in general and on price control in particular. The process of price liberalization makes the inefficient SOEs no longer economically viable and leads to the creation of new and more efficient enterprises [31, 36].

The second dimension is to legalize private economic actors and to eventually create a private sector. Privatization is achieved in two ways: by selling off SOEs (usually starting from small-and medium-sized enterprises) and through the creation of new POEs. Privatization gives companies the freedom in business decision-making, necessary for the transition toward a market economy [31].

In need of economic stabilization, and due to international pressures as they strive for membership in international treaties and trading blocks, post-socialist states have begun to open their national borders to FDI to capture the opportunities afforded by the globalization of the world economy. Internationalization enables these states to obtain much needed capital, technology, as well as managerial know-how. Through innovation diffusion, foreign investors play a catalytic role in the economic transition process for the post-socialist states [31, 37].

#### 2.2.3 Administrative decentralization (Part C in Fig 1)

The Chinese government is a complex and heterogeneous entity: the central and various levels of local government have different power and responsibilities. To encourage local initiatives, the state, since the 1980s, has reformulated the fiscal relationships between the central and local governments, which called a “fiscal contract” system (caizheng baogan) to contract the responsibilities of revenue generation and remittance to local governments. [38]. The central government has decentralized the powers and responsibilities for investment and economic development to provincial and municipal governments. State budgetary allocation of funds no longer contributes significantly to local economic development. The main source of investment has shifted to self-fundraising [39]. The new central–local fiscal contract provided the local governments with primary responsibilities for economic development in their respective jurisdictions and effectively started the decentralization of state power. Specifically, the local governments have been granted greater autonomy over their economies, including the authority to issue business licenses, make investments, transfer land use rights, and coordinate urban developments [40].

Under the new land use system, the municipal government monopolizes the provision of land and plays a significant role in shaping the location of infrastructural investments and the geography of the activities associated with them [2]. After expropriating rural land, the municipal government uses it to construct infrastructure, attract investment, or transfer/lease the land to various economic entities, including manufacturing factories. Moreover, administrative decentralization has made the local governments increasingly gear towards the so-called “entrepreneurship” [41] and led them to spare no effort to create a “friendly” environment to attract investments and increase the local revenues [12]. Therefore, they have established many development zones at specific and advantageous locations, with preferential government policies and professional services, as well as better infrastructure and accessibility, to attract enterprises [9]. This has further enhanced the comparative advantages of the development zones and influenced the location selection by enterprises. Acting as both advocates of local economic activities and regulators of their spatial distribution, the local governments have become an active agent in the spatial restructuring of industrial locations in Chinese cities [40]. In sum, they influence the intra-urban location behavior of manufacturers by affecting their expected costs and profits through the vehicles of land supply, industrial infrastructure, and financial incentives, largely through the establishment of development zones [40].

## 3. Study area, data and methodology

### 3.1 Study area

**Fig 2:**
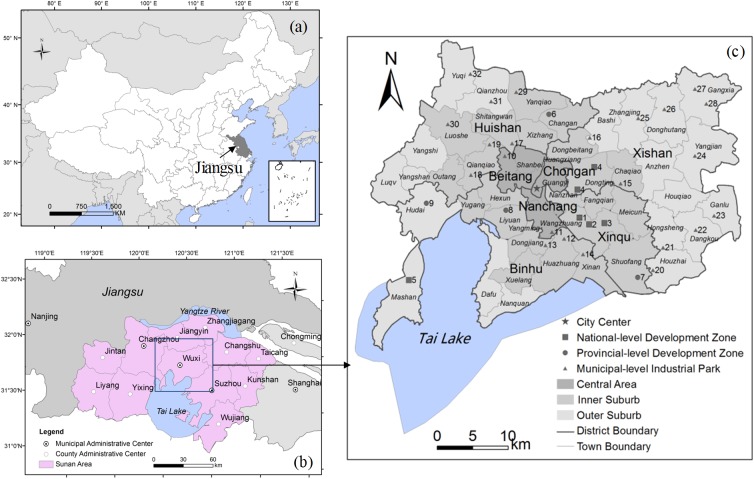
Study area. (a) Jiangsu Province in China, (b) Sunan area in Jiangsu Province, (c) Spatial organization of Wuxi City proper (Note: The numbers represent the development zones and industrial parks; see their names in S1 Table).

With a total land area of 1,295 km^2^ (excluding the Tai Lake), Wuxi consists of 3 central districts and 4 suburban districts (Fig 2). Each district is further divided into communities and townships. For the purpose of this study, we differentiate Wuxi into three zones: the Central Area, the Inner Suburb, and the Outer Suburb. The Central Area is comprised of Chongan, Nanchang and Beitang districts. The division of the Inner and Outer Suburb is based on the contiguous built-up area, which refers to the area where the urban constructions are contiguous distributed. The fractal method [42] is adopted to extract the boundary of the contiguous built-up area of Wuxi by using the TM remote sensing image on July 16, 2013. Outside of the Central Area, the communities or townships with contiguous built-up areas are defined as the Inner Suburb, whereas the rest as the Outer Suburb.

The development zones in Wuxi began to emerge in the early 1990s. By the end of 2013, there existed nine large-scaled development zones in Wuxi, of which five are national-level zones, and four are provincial-level zones (Fig 2). Besides, the municipal governments and township administrations set up many local industrial parks to retain township and village enterprises (TVEs), and accommodate the enterprises relocated from the Central Area. These include 23 municipal-level key industrial parks (see their names in S1 Table).

### 3.2 Data sources

Enterprise-level data were obtained from China’s Second Industrial Census in 1985, and China’s First and Third Economic Census in 2004 and 2013, respectively. As the economic transition in China started after 1978, we chose the 1985 National Second Industrial Census data to show the characteristic of manufacturing location in the early stage of the economic transition. Since the manufacturing in China developed as an extraordinary speed at the early 21^st^ century, we choose the data of China’s first Economic Census in 2004. The data for 2013 is the latest data we can get from China’s three Economic Censuses. All censuses contain enterprise name, full address, industry classification code, year of establishment, ownership, output values, total assets, and the number of employees. In 1985, state owned enterprise (SOE) and collectively-owned enterprise (COE) were still the two predominant ownership types; whereas in 2004 and 2013, there are three additional types: joint-stock (JOE), privately-owned (POE), and foreign-invested (FIE).

Due to the large number of enterprises in Wuxi, we examine those whose output values are 5 million RMB yuan or more in 1985 and 2004, and those whose output values are 10 million RMB yuan or more in 2013. This critical value is used by China National Bureau of Statistics to define enterprises of “designated size”. The data in these three years are fairly comparable, as their shares of enterprises above designated size are consistent in quantity, total asset, and industrial output (Table 1).

**Table 1:** Enterprises above designated size and their share of the city’s totals, 1985, 2004, 2013.

Year	enterprises		total asset		industrial output	
	number	share	Billion RMB*	share	Billion RMB*	share
1985	374	25.1%	6.4	82.1%	8.1	84.3%
2004	4733	23.9%	204.4	86.3%	263.3	92.4%
2013	6045	23.6%	632.9	84.5%	719.6	86.7%

*Source*: Calculated from China’s Second Industrial Census and China’s First and Second Economic Census

* current price.

The enterprise-level data reflect the scale and efficiency characteristics of different types of manufacturing (Table 2). In 1985, although being nearly equal in number of enterprises, the scales of COEs were much smaller than SOEs, as manifested in the number of employment, average industrial output and average number of employees. However, the industrial output value per employee of the SOEs was only slight higher than that of COEs (26.3 vs. 22.2 thousand yuan). This indicates that in the 1980s the production efficiency of the collectively-owned TVEs, which were the bulk of the COEs, was very high.

**Table 2:** Changes of manufacturing enterprises by ownership, 1985, 2004 and 2013.

Year	Attribute	Total	Status of registration (Type of ownership)				
			SOE	COE	JOE	POE	FIE
1985	No. of Enterprises	370	48.4%	51.6%	N/A	N/A	N/A
	No. of Employment(1,000)	323	70.0%	30.0%	N/A	N/A	N/A
	Average Industrial Output Value(million yuan)	21.9	33.2	11.3	N/A	N/A	N/A
	Industrial Output Value per Employee(1,000 yuan)	25.0	26.3	22.2	N/A	N/A	N/A
2004	No. of Enterprises	4700	1.2%	4.8%	13.1%	61.1%	19.8%
	No. of Employment(1,000)	604	5.9%	4.1%	18.5%	37.4%	34.2%
	Average Industrial Output Value(million yuan)	56.0	337.1	51.9	74.1	25.3	122.5
	Industrial Output Value per Employee(1,000 yuan)	435.6	538.9	471.5	409.7	322.1	551.6
2013	No. of Enterprises	6045	0.9%	0.6%	4.7%	68.3%	25.5%
	No. of Employment(1,000)	823	2.3%	0.3%	7.9%	40.1%	49.4%
	Average Industrial Output Value(million yuan)	110.9	574.5	36.1	189.5	50.6	250.0
	Industrial Output Value per Employee(1,000 yuan)	814.3	1651.2	529.6	827.9	632.5	947.8

*Source*: Calculated from China’s Second Industrial Census and China’s First and Second Economic Census.

In 2013, the variations in scale and efficiency among different types of enterprises are even more pronounced. The number of remaining SOEs only accounts for 0.9% of the total enterprises. However, the survived SOE is the most efficient ownership type, as reflected by its industrial output value of 1.6 million yuan per employee. This is twice as high as the industry average (Table 2). FIEs make up only 25.5% of the total enterprises, but provide 49.4% of the manufacturing employment. On average, each FIE produces 250 million yuan of industrial output, significantly more than those of the COEs, JOEs and POEs. The industrial output value per employee of FIEs is 0.9 million yuan, only next to the SOEs, which suggests their high efficiency. Only 4.7% of the enterprises were of joint-stock ownership. Their size and efficiency were both slightly higher than the industry average. Although the POEs account for 68.3% of the total enterprises, they provide only 40.1% of the employment. The POEs and COEs, being similar in average industrial output, average number of employee, and industrial output value per employee, are the smallest in size and the lowest in efficiency among all ownership types. It is clear that the manufacturing industry in Wuxi is now dominated by the large-scale FIEs as well as the large number of small-scale POEs, whereas the SOEs and JOEs are in large scale but small number, and the COEs are out of favor.

### 3.3 Methodology

To explore the spatial shift and the distribution features of enterprises by ownership type, Location Quotient (LQ) analysis is conducted. The LQ for a given activity in area *i* is the ratio of “percentage of the total regional activity in area *i”* to “percentage of the total base in area *i”*. If LQ>1, it indicates a relative concentration of the activity in area *i*, compared to the region as a whole [43]. LQ is quite useful in evaluating the distribution or concentration of manufacturing locations based on administrative boundaries (i.e., community and township, in this study).

As an exploratory point pattern analysis technique, Hot Spot analysis has shown significant advantages in studying industrial location. First, in contrast to LQ, Hot Spot analysis allows us to evaluate the characteristics of manufacturing clusters which may cross administrative units. This may reveal the spatial patterns of manufacturing at a different geographical scale (i.e., development zone). Second, Hot Spots analysis takes manufacturing attributes into consideration, which allows us to examine the spatial pattern of enterprises with high output value. Hot Spot analysis calculates the Getis-Ord Gi* statistic (i.e. *Z*-score) for each enterprise in the dataset. To be a statistically significant hot spot, an enterprise must have a high value and be surrounded by other enterprises with high values as well [44].

$${\text{G}}_{\text{i}}^{*}=\frac{\sum_{j=1}^{n}w_{i,j}x_{j}-\bar{X}\sum_{j=1}^{n}w_{i,j}}{\sqrt[{S}]{\frac{n\sum_{j=1}^{n}w_{i,j}^{2}-{\left(\sum_{j=1}^{n}w_{i,j}\right)}^{2}}{n-1}}}$$ (1)

$$\bar{X}=\frac{\sum_{j=1}^{n}x_{j}}{n}$$ (2)

$$S=\sqrt{\frac{\sum_{j=1}^{n}x_{j}^{2}}{n}-{\left(\bar{X}\right)}^{2}}$$ (3)

Where *x*_*j*_ is the output value for enterprise *j*, *w*_*i*,*j*_ is the spatial weight between enterprise *i* and *j*, *n* is equal to the total number of enterprises, $\bar{x}$ *i*s the mean output value in the whole study area, *S* is the standard deviation of *X*.

For statistically significant positive/negative *Z*-scores, the larger/smaller the *Z*-score is, the more intense the clustering of high/low output values (hot/cold spot) [43]. A *Z*-score of more than 2.58 or less than -2.58 (significant at the 0.01 level) indicates that the distribution of enterprises with high output values or low output values has a clustered pattern, whereas for significance at the 0.05 and 0.1 level, the critical values to be used are 1.96 and 1.65 respectively.

## 4. Changing manufacturing landscape

### 4.1 Decentralization and suburbanization

**Fig 3:**
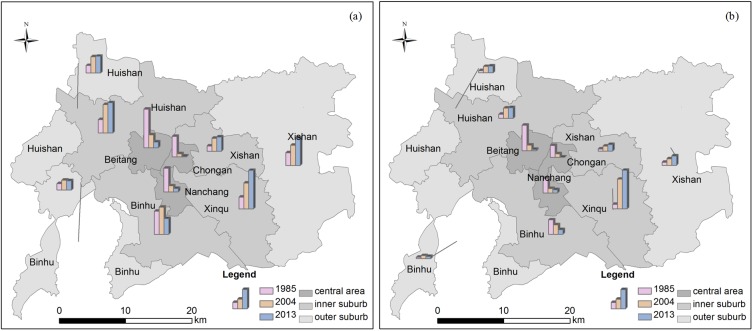
Spatial changes of manufacturing in Wuxi, 1985, 2004 and 2013, by percentage. (a) number of enterprises, (b) industrial output value.

As is shown in Table 3 and Fig 3, manufacturing enterprises exhibited a sharp increase in the Inner Suburb and a relatively slow increase in the Outer Suburb, whereas their shares in the Central Area decreased dramatically. In 1985, enterprises were significantly concentrated in the Central Area. With only 5.5% of the land area in Wuxi, the Central Area hosted 51% of the total enterprises, creating 62% of the industrial production and containing 59% of the employees. However, the proportion of enterprises, output values and employees in the Central Area decreased sharply in 2004 to 14%, 16% and 17% respectively, but increased to 58%, 65% and 58% in the Inner Suburb. By 2013, more than 90% of enterprises were located outside of the Central Area.

**Table 3:** Spatial changes of manufacturing in Wuxi, 1985, 2004 and 2013.

	enterprises%			industrial output value%		
	1985	2004	2013	1985	2004	2013
**Central Area**	50.8	13.6	6.2	62.1	16	5.6
Chongan District	12.6	1.9	0.5	14.1	4.1	0.2
Nanchang District	14.5	3.7	2.1	17.5	5.3	3.9
Beitang District	23.7	8	3.6	30.4	6.6	1.5
**Inner Suburb**	32.8	58	60.7	29.9	65.1	72.3
Binhu District	14.2	16.7	9.7	17.2	11.4	5.6
Huishan District	8.1	17.4	18.5	4.8	12.2	12.7
Xishan District	3.2	8	8.8	2.6	6	7.9
Xinqu District	7.3	15.9	23.7	5.4	35.5	46.1
**Outer Suburb**	16.4	28.4	33	8	18.9	22.1
Binhu District	4	6.1	5.8	1.7	3.7	2.7
Huishan District	4.6	10	10.3	2.7	7.6	8.2
Xishan District	7.8	12.4	16.9	3.6	7.7	11.2

*Source*: Calculated from China’s Second Industrial Census and China’s First and Second Economic Census.

The most profound change is the suburbanization of the newly established enterprises. Among the 2002 enterprises of designated size founded since 2005, 1208 (60.3%) chose to locate in the Inner Suburb and 685 (34.2%) in the Outer Suburb; only 109 (5.5%) of them located within the Central Area, most of which were small-sized enterprises, with the average number of employee less than 50. In addition, the relocation of existing enterprises also contributed to the accelerated decentralization. In 2005, the municipal government of Wuxi issued *Guidance of Industrial Distribution Adjustment in the Central Area*, which identified 116 key manufacturing enterprises (most of them occupied large blocks of land) in the Central Area that needed to be relocated [45].

More importantly, manufacturing enterprises have been unevenly re-distributed across the districts. In 1985, among the three central districts, Beitang District, a traditional industrial district (Fig 2 and Table 3), had the highest concentration of manufacturing, accounting for 30% of the industrial production of Wuxi. About twenty years later, Xinqu District, a suburban area in southeast Wuxi with several designated development zones, became the main cluster of manufacturing. In 2004, it accounted for 35% of the industrial production. By 2013, its share reaches 46%. However, Binhu District (in both the Inner and Outer Suburb), another important agglomeration of manufacturing, had experienced a decline in 2013 compared with 2004. Located by the Tai Lake, the manufacturing plants in Binhu District used to cause severe water pollution. This has led the government to introduce new regulations on its future development: all the polluting factories must move out; no new factories that could cause pollution will be allowed; only a limited number of environmentally-benign or high-tech industries will be permitted. The government encourages city development in areas south of the city center, while shifting manufacturing towards the north. Since 2006, the government has closed and relocated 203 polluting enterprises from Binhu District [45]. As a result, the proportion of manufacturing in the northern districts—Huishan and Xishan District witnessed a slight increase in 2013. This is a clear case of government intervention in manufacturing location with regulatory measures.

### 4.2 Manufacturing geography by ownership

#### 4.2.1 Location Quotient analysis

This section uses LQ to further investigate the variations in the spatial distribution of manufacturing by ownership across community/township. As reflected in Table 4, enterprises of different ownership display distinctive spatial patterns.

**Table 4:** Location quotients for manufacturing enterprises by ownership in Wuxi, 1985, 2004 and 2013.

	District	Community/Township	DZ/IP Level	SOE			COE			JOE		POE		FIE	
				1985	2004	2013	1985	2004	2013	2004	2013	2004	2013	2004	2013
Central Area	Chongan	Chongan		1.7	15.6			4.8		1.1	2.4			2.0	
		Guangyi			4.7	2.7	1.1	1.8	4.0	2.4	2.6			1.2	
	Beitang	Beitang		3.1	10.7	20.4		2.5	7.9	2.0	4.8				
		Shanbei	M		2.2	1.4	1.1					1.1	1.2		
		Huangxiang			1.2				3.3	1.5	2.1				
	Nanchang	Nanchang		2.6	17.4	9.2		2.4	2.7	1.9	5.5			1.2	
		Yangming	M	2.2	5.8	2.4		1.6		2.3	4.0				
Inner Suburb	Binhu	Helie		1.9	7.0	15.5		1.1	2.2	3.5	3.8				
		Liyuan	P	1.4				1.6	3.5	1.7	2.4			1.4	
		Huazhuang	M				1.1					1.3	1.2		
		Dongjiang	M	1.2							1.8	1.2	1.1		
		Yugang					1.1		1.4	2.1	2.6			1.2	
		Xuelang					1.1		1.9				1.2		
		Xinan	M			3.1	1.1				1.1		1.2		
	Huishan	Yanqiao	M			1.7	1.2					1.3	1.1		
		Changan	P				1.2		4.4				1.1		
		Xizhang	P				1.1					1.3	1.1		
		Luoshe	M					1.5				1.2	1.2		
		Shitangwan	M				1.1	1.5				1.2	1.2		
		Outang					1.2					1.3	1.2		
		Qianqiao	M									1.3	1.2		
	Xishan	Dongting	N				1.2							2.1	2.3
		Chaqiao	M				1.1					1.3	1.2		
		Dongbeitang					1.2						1.1	1.9	
	Xinqu	Nanzhan					1.2	3.1	1.7	1.5	1.1				1.4
		Fangqian	N				1.2		1.7		1.1	1.3			1.4
		Wangzhuang	N			2.1	1.2							3.1	3.2
		Meicun					1.2					1.2			1.4
		Shuofang	P				1.2	1.7						1.4	1.8
Outer Suburb	Binhu	Dafu								2.6	1.8		1.2		
		Nanquan					1.2								
		Hudai	P				1.1	1.8	1.6	2.6	1.2		1.2		
		Mashan	N			2.4	1.1	2.7	2.8	1.8	3.9			1.6	1.2
	Huishan	Qianzhou	M				1.1	1.5	2.5			1.1	1.1		
		Yuqi	M				1.1	1.6	1.9			1.2	1.1		
		Yangshi	M				1.2	1.5				1.2	1.2		
		Yangshan										1.5	1.2		
		Luqv	M				1.2					1.5	1.2		
	Xishan	Anzhen	M				1.1					1.3	1.2		
		Houqiao					1.1					1.3	1.2		
		Yangjian	M				1.1			1.8	1.3		1.1		
		Hongsheng	M			1.5	1.2			1.1	2.2	1.2			
		Houzhai				1.5	1.2			1.1	2.2	1.2			
		Ganlu					1.2	1.9	1.2			1.2	1.2		
		Dangkou	M				1.2	1.9	1.2			1.2	1.2		
		Bashi	M				1.2	1.1				1.2	1.1		
		Zhangjing	M				1.1	1.1				1.2	1.1		
		Donghutang					1.1					1.2	1.1		
		Gangxia	M				1.2					1.2	1.1		

Note: The “N” and underline “_” of LQ represent that there exist a national-level development zone in the community/township. The “P” and underline “_” of LQ represent that there exist a provincial-level development zone in the community/township. The “M” and underline “_” of LQ represent that there exist a municipal-level key industrial park in the community/township. *Source*: Calculated from China’s Second Industrial Census and China’s First and Second Economic Census.

It is obvious that in all three years, 1985, 2004 and 2013, the LQs for SOEs reveal a significant concentration in communities of the three central districts. However, over time, the administrative units with higher LQs for SOEs have shifted from the central communities towards the peripheral communities within the three central districts. In addition, several towns in the Inner Suburb and Outer Suburb also show high LQs in 2013. This was reasonable because as the ownership reform proceeded, many SOEs in the Central Area were either transformed to other ownership types or closed; the relocated and newly established SOEs tended to set up plants in the suburbs. There existed 57 SOEs in 2004. By 2013, 26 of them had been either closed or transformed to other ownership types, and 8 had been relocated to the Suburb. All four SOEs founded after 2004, chose to locate in the suburb, with two of them being in the Wuxi High-Tech Industrial Development Zone *(HIDZ)* and Xishan *ETDZ*. The spatial shift of SOEs demonstrates that they enjoy privileges in location selection, and tend to cluster in the national- and provincial-level development zones or areas near the Central Area with better infrastructure. Locating on sites with higher land rents indicates that the location behavior of SOEs is driven by administrative mechanism, rather than by market mechanism.

LQs also show that in 1985, most COEs were concentrated in the towns of the Inner and Outer Suburb. This resulted mainly from the booming development of collectively-owned TVEs in the early 1980s. However, since privatization of the TVEs began in the mid-1990s, the distribution of the remaining COEs has become more concentrated in the Central Area because those in the Suburb are mostly privatized. The JOEs are much more unevenly distributed, with relative concentration in the Central Area and some towns in the Binhu District, which was the administrative suburban area of Wuxi in the 1980s. The SOEs and large-scale COEs were the dominant ownership categories in these areas in the 1980s. Since the ownership reform, most of them were transformed into JOEs. This can be confirmed by the 2004 data, which show that a large proportion of the 245 JOEs founded before 1992, were located in the Central Area (28%) and Binhu District (45%).

**Fig 4:**
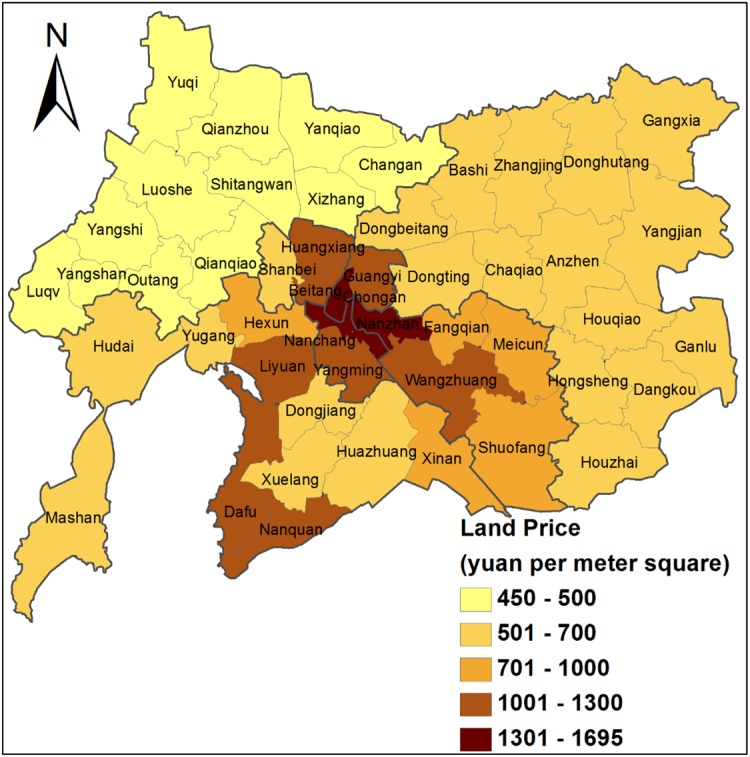
Industrial land price in Wuxi, 2013.

According to the LQs for POEs in 2004 and 2013, it was found that POEs were evenly distributed across the Inner and Outer Suburb, and with less than expected share in the communities/towns within the Xinqu District. On the one hand, this is due mainly to the privatization of collectively-owned TVEs, which were established in these areas. On the other hand, most of the POEs are small-sized enterprises with low-output, which cannot afford the high rents in or near the Central Area and Xinqu District (Figs 2 and 4). Compared with 2004, the concentration areas of POEs in 2013 further shrank in towns close to the Central Area and Xinqu District. This confirms that because of the differential rents by location, the newly established POEs are more likely to locate far away from areas of high land cost or with high property rent.

FIEs are more spatially concentrated than POEs. In 2004, the administrative units with higher LQ were mainly communities and towns that are either in the Central Area or at the locations of national- or provincial-level development zones. However, in 2013 the concentration of FIEs is more intensified. With much reduced concentration in the Central Area, FIEs now mainly concentrate in Dongting, Mashan and the communities and towns in the Xinqu District (Table 4). This indicates that over time, the Central Area is no longer attractive to FIEs because of the limited land availability. By 2013, 47 FIEs had moved out of the Central Area. Among the 476 FIEs founded between 2004 and 2013, only 7 chose to locate at the Central Area. It is also found that the industrial land prices in the administrative units where the FIEs concentrate are much higher than where POEs concentrate. This implies high bidding ability of the FIEs. This spatial pattern of FIEs is also associated with the various newly established national- and provincial-level development zones in these areas, which is explained in the subsequent section.

#### 4.2.2 Hot Spot analysis and the role of development zones

As a specific area designated by government and an ideal locale for manufacturing agglomeration, development zone/industrial park plays a significant role in attracting both domestic and foreign investment. By comparing the distribution of hot spots of enterprises by output values with the location of various development zones, we verify the role of development zones in shaping the new landscape of manufacturing and identify the type of development zones in which manufacturing of each ownership type tends to concentrate.

Within the Municipality of Wuxi, the development zones compete with one another for investment, and their policies toward enterprise are level specific and vary for different types of enterprises. The range of policies contains tax incentives, subsidies, and market access [46]. With better-quality formal institutions and industrial infrastructures, higher-level development zones can also provide enterprises with a more stable and cost-effective environment for their investments [8]. For instance, in national-level development zones, enterprises are usually required to pay a lower corporate tax at 15%, compared with 24% in the provincial-level development zone. In municipal industrial parks, the rate is 30%. Moreover, higher-level development zones also establish their own range of institutions to attract particular types of manufacturing. On the other hand, the average land price and rent in the higher-level development zone are usually higher, which filters (or drives) out some types of manufacturing. Table 5 presents the detailed information for the national- and provincial-level development zones in Wuxi, which demonstrates their differentiated functional orientation, development emphases, and formal institutions.

**Table 5:** National-level and provincial-level development zone in Wuxi, 2013.

Name List	Year of Establishment	Area (km^2^)	Land Price (yuan/m^2^)	Preferential Policies & Entry Requirements
**National-level**				
Wuxi HIDZ	1992	20	1105	• corporate tax rate at 15% • offer an extended tax break to attract high-tech enterprise • attract knowledge- and technology-intensive investments, especially FDI • exclude the existing companies that are of small size and low output • impose restrictions on high energy consumption, serious resource waste, and environment unfriendly and polluting enterprises
Singapore IP	1993	2.31	1045	• same as Wuxi HIDZ
Wuxi EPZ	2002	1.7	770	• same as Wuxi HIDZ
Xishan ETDZ	2003	9.2	635	• corporate tax rate at 15% • mainly for domestic enterprises
Tai Lake NTRA	1992	5.72	525	• corporate tax rate at 15% • strictly prohibit the entry of enterprises that cause pollution or are incompatible with the surrounding scenic landscape
**Provincial-level**				
Huishan EDZ	2002	5.96	490	• corporate tax rate at 24% (15% for high-tech enterprises) • internationally well-known software companies and large-scale enterprises are entitled to lower rent
Shuofang IP	2006	4.53	700	• corporate tax rate at 24% • accept the relocated enterprises, which were originally located in but expelled by the Wuxi HIDZ or other parts of the city that is undergoing urbanization
Liyuan EDZ	1993	2.5	920	• corporate tax rate at 24% • strictly prohibit the entry of enterprises that cause pollution or are incompatible with the surrounding scenic landscape
Wuxi EDZ	2006	2.84	520	• corporate tax rate at 24% • accommodate and re-settle the large-scale enterprises that were originally located by the Tai Lake

Note: HIDZ: High-tech Industrial Development Zone; IP: Industrial Park; EPZ: Export Processing Zone; ETDZ: Economic and Technical Development Zone; NTRA: National Tourism Resort Area; EDZ: Economic Development Zone.

**Fig 5:**
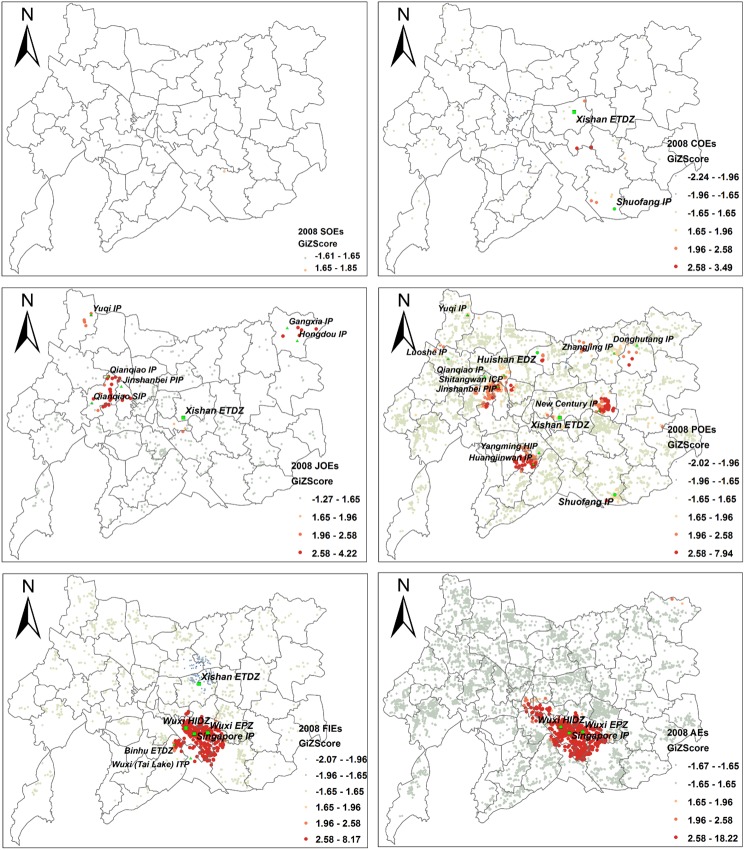
Hotspot analysis of output value of manufacturing enterprises by ownership in Wuxi, 2013 (Note: AE: All Enterprises; HIDZ: High-tech Industrial Development Zone; IP: Industrial Park; EPZ: Export Processing Zone; ETDZ: Economic and Technical Development Zone; NTRA: National Tourism Resort Area; EDZ: Economic Development Zone; PIP: Private Industrial Park; SIP: Supporting Industrial Park; ICP: Industrial Concentration Park; HIP: High-tech Industrial Park; ITP: International Technical Park).

The results of Hot Spot analysis show that distinctive relationships exist between the level of development zone and the ownership type of the hot spots clusters. As is shown in Fig 5, the hot spots of SOEs in 2013 are not significant because of their limited number and scattered distribution. By examining the output of each enterprise, it is found that the SOEs with high output are mainly located in *Wuxi HIDZ*, *Xishan ETDZ* and *Huishan EDZ*, which are national- and provincial-level development zones. Compared with those located in the suburbs, the SOEs in the Central Area do not have high output values. The scarcity of land resources in the Central Area constrained the development of large-scaled SOEs. As a result, most of them chose to relocate to the high-level development zones, thus leaving the relatively small-scaled SOEs in the Central Area (e.g. Oriental Import Car Repair Factory of Wuxi with 40 employees in 2013 still remains in Nanchang District). For the COE, the hot spots are identified in the districts that are home to the national- and provincial-level development zones (i.e., *Xishan ETDZ*, and *Shuofang IP*), while the cold spots are mostly clustered in the Central Area. The significant hot spots of JOE are concentrated in the national-level development zone of *Xishan ETDZ* and six municipal-level key industrial parks (see Fig 5). For the POE, besides the *Xishan ETDZ*, *Huishan EDZ*, and *Shuofang IP*, most of the significant hot spots are scattered in ten municipal-level key industrial parks (Fig 5). However, the hot spots of FIE are heavily concentrated in the national-level development zones (i.e. *Wuxi HIDZ*, *Singapore IP*, and *Wuxi EPZ*). It is also found that the hot spots of all enterprises (AE) combined are geographically leaning toward the hot spots of FIE (Fig 5). This can be explained by the fact that the average industrial output of FIEs is much higher than that of COEs, JOEs and POEs (Table 2), and most of the FIEs that are located in these areas are high-efficiency and high-value added enterprises, such as communication equipment, computers and other electronic equipment manufacturing factories.

*Wuxi HIDZ*, *Singapore IP* and *Wuxi EPZ*, established by the state government, are all globally oriented. Although charging two times of rents than in other development zones, these three national-level zones are still attractive to large-scale FIEs, due to their preferential policies, financial incentives and producer-oriented services and infrastructure. By 2013, 73 of the Fortune 500 companies established factories there. To advance their industrial structure, the zone administrations also establish rules to evaluate the companies that apply to set up factories in these zones, and dispel the existing companies that are in chemical industry, small-sized and with low output.

*Xishan ETDZ* was established in 1992 as a provincial-level development zone, and promoted to a national-level development zone in 2003. Designated mainly for domestic enterprises, it is the location for hot spots of SOEs, COEs, JOEs, and POEs. Interestingly, the cold spots of FIE also cluster in this zone. With the same preferential policies as the *Wuxi HIDZ*, *Singapore IP* and *Wuxi EPZ*, and much lower land prices, *Xishan ETDZ* is attractive to small-sized FIEs.

*Huishan EDZ* is a large-scale provincial-level development zone, mainly accommodating manufacturing activities in the north of the city center. With relatively favorable policies and lowest land price of all the development zones (Table 5), it is appealing to large-scale POEs.

*Shuofang IP* was originally a municipal-level key industry park dominated by TVEs. It was designated as a provincial-level industry park in 2006. Geographically adjacent to the *Wuxi HIDZ*, with quality physical infrastructure, the main purpose of this zone is to accept the relocated enterprises, which were originally located in, but expelled from, the *Wuxi HIDZ* or other parts of the city that is undergoing urbanization. Most of these enterprises are POEs with relatively high output values. This zone also contains a few large-scale TVEs.

The *Tai Lake NTRA* and *Liyuan EDZ*, both located by the Tai Lake, adhere strictly to the environment-friendly regulatory framework for enterprises entry. With more emphasis on environment protection, these two zones are not the location of hot pots of any type of manufacturing.

## 5. Concluding discussions

In this study, the changing manufacturing landscape of Wuxi, a representative city of the Sunan Model, has been investigated from the perspective of enterprise ownership. As well, the changing role of land market and government on the manufacturing landscape reshaping has been examined.

**Fig 6:**
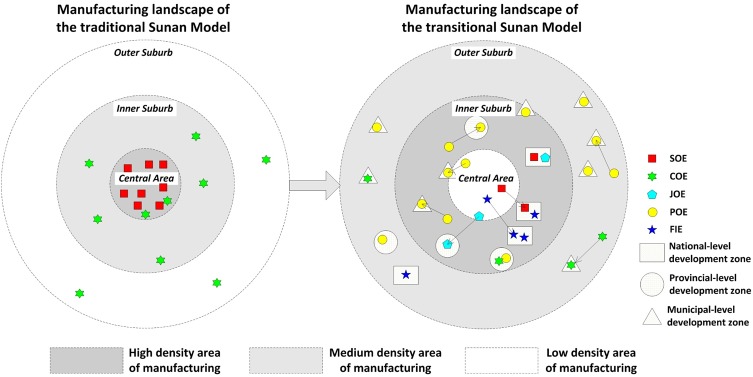
The evolution model of manufacturing landscape in Wuxi.

Since the economic transition, manufacturing in Wuxi has experienced a comprehensive ownership reform, and a new manufacturing landscape has evolved accordingly. That is, manufacturing underwent a distinctive trend of decentralization and suburbanization, as well as an uneven redistribution, agglomerated in various types of development zones. This special process modified the original Sunan Model and led to a transformed model. As Fig 6 illustrates, the state-owned enterprises were initially concentrated in the Central Area. With a small number of them remaining in the Central Area, most of them gravitated to the national-level development zone in the Suburbs over the past two decades. The collectively-owned enterprises have relocated to the town and village industrial parks. A trend of concentration of joint-stock enterprises in the Central Area and Inner Suburb is also observed. Privately-owned enterprises are mainly scattered in the suburbs, while the foreign-invested enterprises are significantly concentrated in the national-level development zone. These observations are supported by the Location Quotient analysis and Hot Spot analysis.

The differentiated spatial patterns of manufacturing by ownership are not only historically rooted, but also market-led and institutionally created through the process of liberalization, privatization and internationalization (see Fig 1). To some extent, the unique spatial pattern of manufacturing is in part the legacy of the original Sunan Model, which is embodied in the spatial distribution of the COEs. It has also become fragmented due to the ownership reform, as many POEs inherited the location characteristics of the small-sized collectively-owned TVEs, and the JOEs bear the geographical features of the SOEs and large-scale COEs. Clearly, the original Sunan Model in Wuxi is phasing out but the influence of the old industrial base and management model still exists, which can still be seen in the location characteristic of the COEs, JOEs, and POEs (see Line 377–390 on Page 16–17). Land marketization is a significant force influencing the restructuring process. The shift from rent-free land use to paid-for land use prompted industrial decentralization and led to a more efficient spatial arrangement of manufacturing. Since the land use system reform began, land price has become the key factor to be considered in an enterprise’s location decision. However, different types of ownership have different ability to negotiate for land use right. The varied ability to bid land (e.g. POEs and FIEs), as well as the differentiated political affiliation with the local government (e.g. SOEs), further contributed to the spatial variations in different ownership types, as depicted in Fig 6.

Different levels of governments played significant, yet varying, roles in the changing manufacturing landscape. The entire manufacturing landscape was affected mostly by the spatial planning and regulatory policies of the municipal government, such as the “Xinqu Development Strategy”, and the policies that “encourage city development in areas south of the city center, while shifting manufacturing towards the north”. The spatial development strategies of the Wuxi municipal government gave each district “industrial development guidance”, through which to control and manipulate the local industrial development process. The manufacturing landscape of the Suburbs was shaped mainly by district government, whose development strategies determined the site selection of each enterprise in local development zones. Being selective in attracting manufacturing investment, the development zone administrations significantly influence the distribution of different ownership enterprises. Through providing preferential policies, the high-level development zones are able to attract the high-efficiency and large-scale enterprises that conform to their industrial development priorities (e.g. FIEs). With high entry bars, the national- and provincial-level development zones filter and exclude the low-efficiency, small-sized, and polluting factories (e.g. POEs). They are also more likely to provide preferential policies to the enterprises that enjoy a close political affiliation with the government (e.g. SOEs).

The findings of this study are relevant to the ongoing enquiry into the intra-urban manufacturing landscape within a broader theoretical context. Although the industrial location theory for the capitalist world cannot be blindly applied to China, the experience found in a city at the forefront of China’s economic transition, such as Wuxi, suggests an interesting local practice wherein the location behavior of the diversified ownership manufacturing has increasingly been steered by the forces of market since the land marketization. Instead of strictly following the market mechanism as in the capitalist world, the inheritance of administrative mechanism in the manufacturing location behavior can still be seen in the Chinese context. However, in contrast to the command economy, under which the state government dictated the allocation of manufacturing through manipulating their operation directly as shown in the original Sunan Model (see Fig 6), the new manufacturing landscape under the economic transition has been characterized by a proactive role played by local governments, which guide the enterprise location decision through a series of formal institutions. On the one hand, we found that the spatial strategies undertaken by local governments in Wuxi have similar elements with other Sunan cities, characterized with providing preferential policies toward high-tech or foreign-invested enterprises; for instance, the case study conducted for Suzhou by Wei, Yuan and Liao [5] revealed similar experiences. On the other hand, we found that the experience of Wuxi is not consistent with the findings of the reconfiguration of industrial districts in Wenzhou, which has gone through a process of delocalization of POEs to globalizing cities and interior cities For example, some of the enterprises have relocated their headquarters and specialized functions to metropolitan areas, especially Shanghai and Hangzhou, and relocated their factories to cities in Inner Monoglia, Henan, and Yunan Provinces etc. [47].

## Supporting information

S1 Table List of development zones/industrial parks. (DOCX)
