# Supplementary material for: Ownership reform and the changing manufacturing landscape in Chinese cities: The case of Wuxi
Source: PLoS One. 2017 Mar 9;12(3):e0173607. doi: 10.1371/journal.pone.0173607 (PMC5344504; doi:10.1371/journal.pone.0173607)
Supplement: S1 Table — (DOCX) [file pone.0173607.s001.docx]

**S1 Table** List of Development Zones/Industrial Parks

| Level | Name List |
| --- | --- |
| National (5) | 1.Wuxi High-tech Industrial Development Zone |
|  | 2.Singapore Industrial Park |
|  | 3.Wuxi Export Processing Zone |
|  | 4.Xishan Economic and Technical Development Zone |
|  | 5.Wuxi Tai Lake National Tourism Resort Area |
| Provincial(4) | 6.Huishan Economic Development Zone |
|  | 7.Wuxi Shuofang Industrial Park |
|  | 8.Liyuan Economic Development Zone |
|  | 9.Wuxi Economic Development Zone |
| Municipal(22) | 10.Jinshanbei Private Industrial Park |
|  | 11.Yangming High-tech Industrial Park |
|  | 12.Binhu Economic and Technical Development Zone |
|  | 13.Huangjinwan Industrial Park |
|  | 14.Wuxi (Tai Lake) International Technical Park |
|  | 15.New Century Industrial Park |
|  | 16.Bashi Industrial Park |
|  | 17.Shitangwan Industrial Concentration Park |
|  | 18.Qianqiao Supporting Industrial Park of Huishan Economic Development Zone |
|  | 19.Qianqiao Industrial Park |
|  | 20.Houzhai Industrial Park |
|  | 21. Machine Photoelectric Equipment Manufacturing Industrial Park |
|  | 22.Ehu Industrial Park |
|  | 23.Ganlu Industrial Park |
|  | 24.Yangjian Industrial Park |
|  | 25.Zhangjing Industrial Park |
|  | 26.Donghutang Industrial Park |
|  | 27.Gangxia Industrial Park |
|  | 28.Hongdou Industrial Park |
|  | 29.Yanqiao Industrial Park |
|  | 30.Luoshe Industrial Park |
|  | 31.Qianzhou Industrial Park  32.Yuqi Industrial Park |
